# Prenatal Opioid Use Disorder and the Risk of Congenital Anomalies in Offspring: A Population‐Based Study

**DOI:** 10.1002/bdr2.2456

**Published:** 2025-02-20

**Authors:** Kaylee Ramage, Jennifer Yee, Sebastian Srugo, Julian Little, Shiliang Liu

**Affiliations:** ^1^ Department of Community Health Sciences University of Calgary Calgary Canada; ^2^ School of Public Health, san Diego State University San Diego USA; ^3^ Centre for Surveillance and Applied Research, Public Health Agency of Canada Ottawa Canada; ^4^ School of Epidemiology and Public Health University of Ottawa Ottawa Canada

**Keywords:** birth defects, congenital anomaly, congenital heart disease, congenital microcephaly, opioid use

## Abstract

**Objective:**

To examine whether prenatal opioid use disorder (OUD) diagnosis is associated with the risk of congenital anomalies (CAs) in offspring.

**Methods:**

We conducted a population‐based study of mother–newborn dyads comprising.

4143 761 births delivered in Canada from 2006 to 2021. We used robust Poisson regression to examine the association between prenatal OUD diagnosis and risk of non‐chromosomal CAs, adjusted for maternal age, parity, multiple gestation, co‐morbidities (including mental health disorders, chronic illnesses and other substance use disorders), and infant sex.

**Results:**

We identified a total of 21, 638 births to persons who were diagnosed with prenatal OUD and 65, 992 (159.3 per 10,000) newborns with CAs. The overall risk of CAs was 2.3 times higher in infants born to birthing persons with a diagnosis of OUD (95% CI 2.2, 2.5). Compared to those without OUD diagnoses, births to persons with a diagnosis of OUD had a higher risk of specific types of congenital microcephaly (aRR 5.2, 95% CI 4.1, 6.6), cleft palate (RR 4.8, 95% CI 3.7, 6.1), pulmonary valve atresia with intact ventricular septum (aRR 2.7, 95% CI 1.1, 6.7), and atrial septal defect (aRR 3.1, 95% CI 2.8, 3.5), among others. In particular, infants born to those with an OUD diagnosis had a 1.8 (95% CI 1.4, 2.3)‐fold increased risk of having severe congenital heart disease.

**Conclusion:**

Our findings suggest an association between prenatal OUD diagnosis and certain CAs in the offspring. Future research is necessary to better understand the role of socio‐demographic factors on these associations.

## 1 Background

Perinatal maternal substance use has become of significant public health importance, particularly in view of changes in substance use policy and the high level of morbidity and mortality related to opioid use in Canada (Fischer 2023). Given their increasing use in the Canadian population, the effects of prenatal opioid exposure on fetal and child development are of particular concern.

Opioid use in Canada has been steadily increasing in the general population (Belzak and Halverson 2018). Data from the 2019 Canadian Alcohol and Drugs Survey revealed that 14% of Canadians aged 15 years and older reported having used opioids in the previous year compared to 12% reported in 2017 (Health Canada 2021). Increasing opioid use has also been reported in pregnant populations in Canada (Corsi et al. 2020). An examination of opioid‐dependent women in Ontario, Canada between 2002 and 2014 found that the number of infants born to women with opioid abuse and dependence had increased from 46 to 800 per year during this time (Brogly et al. 2017). Similarly, an analysis of labor and delivery hospitalizations in the U.S.A. from 2010 to 2017 showed an increase in maternal opioid‐related diagnosis rates at delivery from 3.5 per 1000 hospitalizations in 2010 to 8.2 per 1000 hospitalizations in 2017 (Hirai et al. 2021).

Opioid use in pregnancy has been associated with a variety of adverse outcomes for the developing fetus, including structural effects within the brain, neonatal abstinence syndrome (NAS), preterm birth, low birthweight, and small‐for‐gestational‐age births (Corsi et al. 2020; Merhar et al. 2021; Nørgaard et al. 2015; Patrick et al. 2015; Turner et al. 2015). Furthermore, prenatal opioid exposure has been reported to be associated with several types of congenital anomalies (CAs) (Viteri et al. 2015). A 2017 systematic review (Lind et al. 2017) of 68 studies found that prenatal opioid exposure was associated with higher rates of oral clefts, ventricular septal defects, atrial septal defects, and clubfoot; however, the authors noted that considerable uncertainty remains regarding the teratogenicity of opioids given the variability in study design, poor data quality, and inadequate consideration of potential confounders in the included studies. One ecological study also found an association between opioid prescription rates and trends in gastroschisis (Short 2019).

Given that the growing rate of prenatal opioid use disorder (OUD) in the Canadian population is of public health concern, it is important to investigate the relationship of this exposure to CAs and to provide population‐based evidence to fill existing gaps in the literature. To respond to this need, we assessed the association between prenatal OUD diagnosis and the risk of CAs in Canada.

## 2 Methods

This study adheres to the STROBE reporting guidelines. Using data from the Discharge Abstract Database (DAD), we conducted a population‐based analysis of livebirths and stillbirths (including late pregnancy terminations) occurring in hospitals in Canada (excluding Quebec) between April 1, 2006 to March 31, 2021. The DAD is maintained by the Canadian Institute for Health Information (CIHI) and includes records of hospital labor and delivery for all mothers and babies at ≥ 20 weeks gestation in Canada (excluding Quebec). Within CIHI, the DAD records are collated by trained medical record personnel using standardized definitions and include information on maternal and newborn characteristics (e.g., gestational age, plurality, birthweight) and maternal and newborn diagnoses (up to 25 diagnostic fields) and interventions (up to 20 procedure fields) noted during the labor and delivery hospitalization. Validation studies have shown data from the DAD to have high sensitivity and specificity in the coding of major perinatal procedures and outcomes (Joseph et al. 2009), especially CAs (Public Health Agency of Canada 2013), and this database has previously been used for perinatal research (Liu et al. 2021; Plouffe et al. 2023; Ramage et al. 2019).

Liveborn infants were linked to maternal medical records using a unique, CIHI‐assigned maternal‐newborn identifier. Stillbirths (including late‐term pregnancy terminations) were linked through a previously validated algorithm (Liu et al. 2019) to the maternal childbirth medical records. Maternal and infant characteristics were captured in the DAD, including maternal age (categorized as < 20 years, 20–24 years, 25–29 years, 30–34 years, 35–39 years, and 40–49 years), parity (categorized as first, second, third, or higher order child, and missing values), newborn sex, residence (rural vs. urban), chronic conditions or illnesses, prenatal use of other specified/unspecified drugs or medications (e.g., alcohol, tobacco, and psychostimulants), pregestational diabetes mellitus, and maternal non‐chromosomal anomalies; these were included in the analysis as covariates (Liu et al. 2015). To account for non‐independence in the dataset (i.e., multiple deliveries by the same birthing person over the study period), we included only the first delivery in the dataset for each birthing person.

The full list of International Statistical Classification of Diseases and Related Health Problems, 10th Revision, Canadian edition (ICD‐10‐CA) codes used to classify maternal and infant exposures are available in Tables S1 and S2, respectively. Birthing persons with a diagnosis of prenatal OUD were identified by maternal codes F11 (opioid‐related disorders), T40.0 (poisoning by, adverse effect of, and underdosing of narcotics and psychodysleptics), or R78.1 (finding of opioid in blood) or newborn code P96.1 (neonatal withdrawal symptoms from maternal use of drugs of addiction or “neonatal abstinence syndrome”), which aligns with other indicators of prenatal opioid use using the DAD (Camden et al. 2021). Diabetes mellitus, maternal pain‐related diseases, and major CAs were included in the analysis as covariates. Maternal conditions/illnesses were coded using the ICD‐10‐CA, including lupus, epilepsy, migraine, severe obesity, and use of other substances. CAs in infants (up to 1 year old) identified after birth hospital discharge through a pre‐verified follow‐up linkage algorithm were added to the birth outcome (Liu and Wen 1999).

Diagnoses of CAs among newborns were also coded using the ICD‐10‐CA, including those of the central nervous system (i.e., neural tube defects, microcephaly), heart, kidney (i.e., renal agenesis, cystic kidney disease), and so on. Newborns with Down syndrome and other chromosomal anomalies (*n* = 9129) identified were excluded from the analysis. A full description of the ICD‐10‐CA codes used is presented in Table S2.

We first conducted descriptive statistics to calculate the prevalence of newborns with CAs by maternal characteristics. The prevalence of specific CAs was expressed as the number of cases per 10,000 births. For opioid‐exposed newborn groups, we estimated the crude and adjusted risk ratios (aRRs) with 95% confidence intervals (CIs) for each selected type of CAs using a Poisson distribution with robust standard error estimates to facilitate model convergence. In our multivariable analyses, we adjusted for covariates including maternal age, parity, multiple birth, infant sex, co‐morbidities (e.g., lupus, migraine, obesity, maternal chronic conditions including lupus, migraine, arthritis, or cancer (as a proxy for pain management)), maternal mental health disorders (mood or anxiety disorder, bipolar disorder, schizophrenia), rural (versus urban) residence, and maternal use of other substances or drugs including tobacco, alcohol, cannabinoids, psychostimulants, and other illicit drugs. Each specific type of CA was estimated individually using a crude risk ratio and aRRs using multivariate regression.

Analyses were also conducted to examine issues related to the robustness of the results to unmeasured confounding using E‐value methodology (Van der and Ding, 2017). The E‐value is a measure related to evidence for causality and represents the minimum strength of association that an unmeasured confounder would need to have with both a fetal exposure and birth with a CA, conditional on the confounders in the regression model, to fully explain the observed association (Van der and Ding, 2017).

## 3 Results

Our study population comprised 4,143,761 deliveries, including first live births or stillbirths delivered at ≥ 22 weeks' gestation at hospitals in Canada (except Quebec) from fiscal year 2006/07 to 2020/21. Table 1 describes the characteristics of our study population. Overall, 0.52% of deliveries in our sample were to birthing persons with OUD diagnoses (*n* = 21, 638). Compared to those without diagnosed opioid used disorder, birthing persons diagnosed with OUD were younger and had higher levels of concurrent mental health disorders (6.7% vs. 0.9%), tobacco use disorder (3.9% vs. 0.5%), alcohol use disorder (2.6% vs. 0.1%), and use of psychostimulants (10.9% vs. 0.1%). Over the study period, the proportion of deliveries to birthing persons with diagnosed OUD increased (20.5% occurred between 2006 and 2011 compared to 41.9% between 2016 and 2021) compared to the relatively constant proportion of deliveries to those without diagnosed prenatal OUD over the study period.

**TABLE 1:** Characteristics according to presence of prenatal opioid use disorder (OUD) diagnosis in Canada excluding Quebec, 2006–2021.

Characteristic	Birthing person diagnosed with OUD (%)		Birthing person not diagnosed with OUD (%)	
Total	21,638	100.0	4,122,123	100.0
Age at delivery (year)				
< 20	916	4.2	136,445	3.3
20–24	4937	22.8	542,957	13.2
25–29	7151	33.1	1,163,965	28.3
30–34	5633	26.0	1,407,261	34.1
35–49	3001	13.9	871,495	21.1
Parity				
1st child	8128	37.6	1,466,783	35.6
2nd child	5281	24.4	1,131,935	27.4
≥ 3rd child	3493	16.1	729,204	17.7
Missing data^a^	4736	21.9	794,201	19.3
Multiple birth				
Yes	431	2.0	118,959	2.9
No	21,207	98.0	4,006,164	97.1
Infant sex				
Male	11,654	53.9	2,113, 412	1.3
Female	9984	46.1	2,008,711	48.7
Mental health disorder^b^				
Yes	1446	6.7	35,827	0.9
No	20,192	93.3	4,086,296	99.1
Substance use disorder				
Tobacco	845	3.9	20,937	0.5
Alcohol	545	2.6	4194	0.1
Psychostimulants^c^	2359	10.9	4568	0.1
Miscellaneous^d^	3791	17.5	124,979	3.0
Maternal chronic condition or illness				
Epilepsy	78	0.4	4718	0.1
Lupus	35	0.2	3357	0.1
Pre‐gestational diabetes	156	0.7	11,379	0.3
Severe obesity	436	2.0	75,884	1.8
Migraine	29	0.1	1354	0.3
Other pain‐related diagnoses^e^	160	0.7	18,188	0.4
Maternal residence				
Rural	5014	23.3	668,609	16.2
Urban	16,624	76.7	3,453,514	83.8
Birth period				
2006/07–2010/11	4443	20.5	1,356,178	32.9
2011/12–2015/16	8127	37.6	1,395,957	33.9
2016/17–2020/21	9068	41.9	1,369,988	33.2

^a^ Missing values for parity are treated as a specific category rather than imputing data.

^b^ Depression, stress, anxiety, bipolar disorder, schizophrenia, delusional disorder, and personality disorders.

^c^ Psychostimulants include cocaine and other stimulants.

^d^ Includes inhalant, cannabinoids, psychoactive, or psychotropic substances and unspecified drugs.

^e^ Arthritis, cancer, or major birth defects.

Table 2 describes the association between prenatal OUD diagnosis and CAs. Overall, 1.59% of infants were diagnosed with at least one type of CA (*n* = 65, 992), yielding an overall prevalence of 159.3 per 10, 000 births. Atrial septal defect was the most common type of CAs (59.6 per 10,000 births), followed by ventricular septal defect (32.8 per 10,000 births) and hypospadias (30.2 per 10,000 births). All other CAs examined in this study had risks less than 10 cases per 10, 000 births. Compared to infants born to birthing persons who were not diagnosed with OUD, those infants born to birthing persons with diagnosed OUD were 2.34 times (95% CI: 2.19, 2.56) as likely to have a CA. aRRs were highest for congenital microcephaly (aRR: 5.20, 95% CI: 4.08, 6.62), cleft palate (aRR: 4.76, 95% CI: 3.71, 6.11), and atrial septal defect (aRR: 3.14, 95% CI: 2.84, 3.47). As well, those infants born to birthing persons with diagnosed prenatal OUD were also significantly more likely to have neural tube defects (aRR: 1.63, 95% CI: 1.11–2.40), coarctation of the aorta (aRR: 2.22, 95% CI: 1.50, 3.38), Tetralogy of Fallot (aRR: 1.97, 95% CI: 1.23–3.16), pulmonary valve atresia with intact ventricular septum (aRR: 2.67, 95% CI: 1.06, 6.70), left ventricular outflow tract obstruction (aRR: 2.01, 95% CI: 1.45–2.78), conotruncal defect (aRR: 1.87, 95% CI: 1.35–2.60), ventricular septal defect (aRR: 2.51, 95% CI: 2.16, 2.92), gastroschisis (aRR: 1.34, 95% CI: 1.22, 2.20), cystic kidney disease (aRR: 1.90, 95% CI: 1.33, 2.74), and hypospadias (aRR: 1.23, 95% CI:1.03, 1.50).

**TABLE 2:** Association between prenatal opioid use disorder (OUD) diagnosis and selected congenital anomalies in Canada excluding Quebec, 2006–2021.

Type or subtype of anomaly	Total		Delivery to birthing person diagnosed with OUD (*N* = 21,638)		Delivery to birthing person not diagnosed with OUD (*N* = 4,122,123)		Risk ratio (RR, 95% CI)	
	Total N	Per 10,000	Total N	Risk per 10,000 total births	Total N	Risk per 10,000 total births	Unadjusted	Adjusted*
All specified anomalies	65,992	159.3	957	442.3	65,035	157.8	2.80 (2.63–2.99)	2.34 (2.19–2.50)
Neural tube defects	3207	7.3	29	13.4	2998	7.3	1.84 (1.28–2.66)	1.63 (1.11–2.40)
Congenital microcephaly	2323	5.6	93	43.0	2230	5.4	7.94 (4.46–9.78)	5.20 (4.08–6.62)
Critical congenital heart defects	7561	18.2	80	37.0	7481	18.1	2.04 (1.63–2.54)	1.79 (1.42–2.26)
Hypoplastic left heart syndrome (HLHS)	904	2.2	9	4.2	895	2.2	2.52 (1.77–3.59)	1.79 (0.89–2.53)
Coarctation of the aorta (CoA)	2364	5.7	28	12.9	2336	5.7	1.92 (0.99–3.69)	2.22 (1.50–3.38)
Aortic valve stenosis (AoS)	695	1.7	6	2.8	689	1.7	1.66 (0.74–3.71)	1.18 (0.50–2.81)
Tetralogy of Fallot (TOF)	1723	4.2	19	8.8	1704	4.1	2.12 (1.35–3.34)	1.97 (1.23–3.16)
d‐transposition of great arteries (DTGA)	1313	3.2	11	5.0	1302	3.2	1.61 (0.89–2.91)	1.50 (0.81–2.77)
Double outlet right ventricle	885	2.1	6	2.8	879	2.1	1.26 (0.58–2.90)	1.05 (0.45–2.41)
Persistent truncus arteriosus (PTA)	377	0.9	5	2.3	372	0.9	3.11 (0.99–9.72)	2.67 (1.06–6.70)
Total anomalous pulmonary venous return (TAPVR)	471	1.1	6	2.8	465	1.1	2.52 (1.04–6.08)	1.74 (0.73–6.19)
Left ventricular outflow tract obstruction (LVOTO)	3469	8.4	39	18.0	3430	8.3	2.31 (1.47–2.76)	2.01 (1.45–2.78)
Conotruncal defect	3699	8.9	39	18.0	3660	8.9	2.03 (1.48–2.78)	1.87 (1.35–2.60)
Right ventricular outflow tract obstruction (RVOTO)	602	1.5	6	2.8	596	1.4	1.92 (0.86–4.29)	1.80 (0.78–4.18)
Ventricular septal defect (VSD)	13,579	32.8	200	92.4	13,379	32.5	2.85 (2.48–3.27)	2.51 (2.16–2.92)
Atrial septal defect (ASD)	24,682	59.6	460	212.6	24,222	59.9	3.62 (3.30–3.97)	3.14 (2.84–3.47)
Gastroschisis	1436	3.5	17	7.9	1419	3.5	2.24 (1.39–3.61)	1.34 (1.22–2.20)
Cystic kidney disease	2991	7.2	33	15.3	2958	7.2	2.13 (1.51–3.00)	1.90 (1.33–2.74)
Cleft palate	2746	6.6	78	36.0	2668	6.5	5.57 (4.45–6.98)	4.76 (3.71–6.11)
Cleft lip ± cleft palate	3943	9.5	29	13.4	3914	9.5	1.41 (0.98–2.03)	1.11 (0.76–1.63)
Esophageal atresia ± tracheoesophageal fistula	1122	2.7	13	6.0	1109	2.7	2.23 (1.29–3.86)	1.79 (0.99–3.22)
Hypospadias	12,513	30.2	88	40.7	12,425	30.1	1.35 (1.09–1.66)	1.23 (1.03–1.60)
Transverse limb deficiency	1389	3.4	13	6.0	1376	3.3	1.80 (1.04–3.11)	1.26 (0.71–2.25)
Renal agenesis & stenosis	2064	5.0	8	3.7	2056	5.0	0.74 (0.37–1.48)	0.64 (0.31–1.28)
Anorectal atresia & stenosis	1669	4.0	16	7.4	1633	4.0	1.87 (1.14–3.05)	1.60 (0.95–2.70)

* Adjusted risk ratio (aRR) for all or specified subtype of anomaly is estimated after adjusting for maternal age, parity, multiple gestation, chronic condition or illness, pregestational diabetes, substance use disorders, including tobacco, alcohol, cannabinoids, psychostimulants, inhalant, and psychoactive or psychotropic substances, rural (versus urban) residence, newborn sex, and 5‐year period of birth using the categories presented in Table 1.

E‐values for associations between specified CAs and maternal prenatal diagnosis of OUD are included in Table S3. These E‐values suggested that relatively strong confounding assumptions would be needed to eliminate the associations between prenatal exposure to maternal OUDs and specific CAs in offspring. For example, E‐values for the association between maternal prenatal OUD and the risk of congenital microcephaly (*E* = 9.87 and lower 95% confidence bound 7.62), the risk of cleft palate (8.89 and 6.88, respectively), the risk of atrial septal defect (5.73 and 5.13, respectively), the risk of ventricular septal defect (4.46 and 3.74, respectively), and the risk of coarctation of the aorta (3.87 and 2.37, respectively).

## 4 Discussion

Overall, our analysis demonstrates that diagnosed maternal OUD may be associated with certain CAs in infants, highlighting the need for continued surveillance and support to mitigate maternal substance use disorder, especially with increasing trends of opioid use in Canada. The prevalence of diagnosed prenatal OUD was relatively low (0.52%), but likely under‐ascertained (Cook 2022). These exposures, although affecting only a small proportion of pregnancies/births, have the potential to affect thousands of births across Canada, reflect possibly life‐threatening and long‐lasting health outcomes, and are of serious public health concern.

Our study examined the relationship between prenatal OUD diagnosis and CAs; recent studies focusing on maternal opioid use and CAs have examined prescription opioid exposure by trimester (Wen et al. 2021), NAS and CAs (Bhatt et al. 2022), the treatment of OUD during pregnancy, the risk of CAs (Suarez et al. 2022, 2024), and other adverse neonatal outcomes (Piske et al. 2021). A meta‐analysis of cohort studies examining opioid exposure during pregnancy and the risk of congenital malformations found that opioid exposure during pregnancy was associated with a 30% increased risk of congenital malformations overall (Wang et al. 2022). However, several studies have found no such association (Greig et al. 2012; Källén et al. 2013) and few studies have examined the risk of CAs associated with diagnosed prenatal opioid disorder. In a small cohort study of Canadian women being treated for OUD (*n* = 45 mother–baby dyads), 17.8% of neonates were identified as having a CA (including bilateral hydroceles, Tetralogy of Fallot, right‐hand malformation, hypospadias, ankyloglossia, and ventricular septal defect, among others) (Miller et al. 2019). It is possible that other factors associated with OUD can influence CAs and other fetal outcomes, such as opioid substitution treatment, frequency and severity of prenatal opioid use, and socio‐economic status. Future studies should examine the impact of these factors on the relationship between prenatal OUD diagnosis and CAs.

In our study, there was potential for non‐differential exposure misclassification, representing only diagnosed cases of maternal OUD during pregnancy, with babies affected by CAs being more likely to be coded as having exposure to a substance in delivery records than those without. There is also the possibility of differential classification of the outcome, in which birthing persons diagnosed with OUD during delivery may be more likely to have their infants coded for a CA. Similarly, only 0.52% of births in our study cohort were identified as being to those with a prenatal OUD diagnosis when other studies have estimated that diagnosed OUD is present in 0.82% of labor and delivery hospitalizations (Hirai et al. 2021).

This study had both strengths and limitations. Our study responds to the need to generate more evidence on the impacts of OUD during pregnancy. Although CAs are generally considered rare events, our study design using a large, population‐based, administrative dataset with strong internal validity (Joseph et al. 2009) allowed us to identify nearly all cases of CAs during the labor and delivery hospitalizations and neonatal readmissions over the study period. Furthermore, it provided us with statistical power to estimate associations and to control for potential confounders. The E‐values obtained from our sensitivity analysis also suggest that unmeasured confounding is unlikely to account for the associations with specific CAs. Our study data also provide updated estimates of diagnosed OUD during pregnancy across the study period.

Because maternal substance use can be highly stigmatized, it is important to recognize potential limitations of the measurement of maternal substance use. First, our study relied on the DAD for the measurement of both exposure and outcome, and OUD may have been missed or only reported as OUD even if polydrug use was present. Second, our use of data from the DAD only at the labor and delivery hospitalization affected examination of (i) timing of exposure, as we focused only on the labor and delivery hospitalization as the sole point of exposure measurement and we were not able to estimate dose or frequency of exposure, which might also have affected fetal development, and (ii) outcome measurement, as CAs that were not identified at this time were not captured in our analysis. Third, as this study involved secondary analysis of an administrative dataset, it is possible that exposures or outcomes could be miscoded (e.g., it is possible that the use of P96.1 to code maternal opioid exposure may have caused some false positives as some neonates with withdrawal symptoms who are coded with P96.1 may be actually due to other “drugs of addiction” besides opioids). Finally, our use of an administrative database limited our inclusion of potential confounders (e.g., socioeconomic status) and treatment for OUD in our analyses. Although our sensitivity analysis indicated that a high level of unmeasured confounding would be necessary to fully nullify the associations observed in our study (indicating the robustness of our findings) (Van der and Ding, 2017), caution should be taken when interpreting these results. Prospective studies are needed to examine these potential confounders and the effects that timing, dose, or frequency of prenatal opioid use might have on birth outcomes (Saleh Gargari et al. 2012). Future studies should also examine the impact of prenatal exposure to maternal opioid treatment on the development of CAs.

## 5 Conclusions

Our study examined the relationship between prenatal OUD diagnosis and the risk of CAs in the offspring. Our findings suggest that prenatal OUD diagnosis may be associated with certain CAs in newborns; however, future analysis examining the impact of substance use patterns, substance use treatment, and other potential confounders is necessary and can provide estimates that more accurately represent the true relationship between prenatal opioid exposure and CAs. Our study contributes important information for clinicians and other service providers to support healthy pregnancy and fetal development. In accordance with recommendations from the Society of Obstetricians and Gynecologists of Canada (Ordean et al. 2017), clinicians should be prepared to screen birthing persons for substance use during pregnancy, provide guidance regarding the risks of substance use during pregnancy, and make appropriate referrals.

## Author Contributions

Liu had full access to all of the data in the study and takes responsibility for the integrity of the data and the accuracy of the data analysis.

## Ethics Statement

The authors have nothing to report.

## Conflicts of Interest

The authors declare no conflicts of interest.

## Supporting information


**Table S1.** International Classification of Disease Codes for maternal risk factors, conditions or illnesses.
**Table S2**. International Classification of Disease Codes: Infant Congenital Anomalies.
**Table S3**. E‐values expressing the required risk ratio for any unmeasured confounder to overcome the observed association of specific congenital anomalies with prenatal diagnosis of opioid use disorder.

## Data Availability

The data that support the findings of this study are available from Canadian Institute for Health Information. Restrictions apply to the availability of these data, which were used under license for this study. Data are available from the author(s) with the permission of Canadian Institute for Health Information.

## References

[bdr22456-bib-0001] Belzak, L. , and J. Halverson . 2018. “Evidence Synthesis ‐ The Opioid Crisis in Canada: A National Perspective.” Health Promotion and Chronic Disease Prevention in Canada: Research, Policy and Practice 38, no. 6: 224–233. 29911818 10.24095/hpcdp.38.6.02 PMC6034966

[bdr22456-bib-0002] Bhatt, P. , C. Ampem‐Darko , G. A. Cudjoe , et al. 2022. “Association Between Neonatal Abstinence Syndrome and Congenital Anomalies in the United States.” American Journal of Perinatology 41: e1023–e1029. 10.1055/s-0042-1759864. 36572036

[bdr22456-bib-0003] Brogly, S. B. , S. Turner , K. Lajkosz , et al. 2017. “Infants Born to Opioid‐Dependent Women in Ontario, 2002‐2014.” Journal of Obstetrics and Gynaecology Canada: JOGC = Journal d'obstetrique et Gynecologie Du Canada: JOGC 39, no. 3: 157–165. 10.1016/j.jogc.2016.11.009. 28343557

[bdr22456-bib-0004] Camden, A. , J. G. Ray , T. To , T. Gomes , L. Bai , and A. Guttmann . 2021. “Prevalence of Prenatal Opioid Exposure in Ontario, Canada, 2014‐2019.” JAMA Network Open 4, no. 2: e2037388. 10.1001/jamanetworkopen.2020.37388. 33595660 PMC7890532

[bdr22456-bib-0005] Cook, J. L. 2022. “Epidemiology of Opioid Use in Pregnancy.” Best Practice & Research. Clinical Obstetrics & Gynaecology 85: 12–17. 10.1016/j.bpobgyn.2022.07.008. 36045026

[bdr22456-bib-0006] Corsi, D. J. , H. Hsu , D. B. Fell , S. W. Wen , and M. Walker . 2020. “Association of Maternal Opioid Use in Pregnancy With Adverse Perinatal Outcomes in Ontario, Canada, From 2012 to 2018.” JAMA Network Open 3, no. 7: e208256. 10.1001/jamanetworkopen.2020.8256. 32725246 PMC12064095

[bdr22456-bib-0007] Fischer, B. 2023. “The Continuous Opioid Death Crisis in Canada: Changing Characteristics and Implications for Path Options Forward.” Lancet Regional Health 19: 100437. 10.1016/j.lana.2023.100437. 36950034 PMC10025405

[bdr22456-bib-0008] Greig, E. , A. Ash , and A. Douiri . 2012. “Maternal and Neonatal Outcomes Following Methadone Substitution During Pregnancy.” Archives of Gynecology and Obstetrics 286, no. 4: 843–851. 10.1007/s00404-012-2372-9. 22584603

[bdr22456-bib-0009] Health Canada . 2021. “Canadian Alcohol and Drugs Survey (CADS): Summary of results for 2019 [Surveys].”

[bdr22456-bib-0010] Hirai, A. H. , J. Y. Ko , P. L. Owens , C. Stocks , and S. W. Patrick . 2021. “Neonatal Abstinence Syndrome and Maternal Opioid‐Related Diagnoses in the US, 2010‐2017.” JAMA 325, no. 2: 146–155. 10.1001/jama.2020.24991. 33433576 PMC7804920

[bdr22456-bib-0011] Joseph, K. S. , J. Fahey , and Canadian Perinatal Surveillance System . 2009. “Validation of Perinatal Data in the Discharge Abstract Database of the Canadian Institute for Health Information.” Chronic Diseases in Canada 29, no. 3: 96–100. 19527567

[bdr22456-bib-0012] Källén, B. , N. Borg , and M. Reis . 2013. “The Use of Central Nervous System Active Drugs During Pregnancy.” Pharmaceuticals (Basel, Switzerland) 6, no. 10: 1221–1286. 10.3390/ph6101221. 24275849 PMC3817603

[bdr22456-bib-0013] Lind, J. N. , J. D. Interrante , E. C. Ailes , et al. 2017. “Maternal Use of Opioids During Pregnancy and Congenital Malformations: A Systematic Review.” Pediatrics 139, no. 6: e20164131. 10.1542/peds.2016-4131. 28562278 PMC5561453

[bdr22456-bib-0014] Liu, S. , J. Evans , A. Boutin , et al. 2021. “Time Trends, Geographic Variation and Risk Factors for Gastroschisis in Canada: A Population‐Based Cohort Study 2006‐2017.” Paediatric and Perinatal Epidemiology 35, no. 6: 664–673. 10.1111/ppe.12800. 34472132 PMC9291817

[bdr22456-bib-0015] Liu, S. , J. Evans , A. J. MacFarlane , et al. 2019. “Association of Maternal Risk Factors With the Recent Rise of Neural Tube Defects in Canada.” Paediatric and Perinatal Epidemiology 33, no. 2: 145–153. 10.1111/ppe.12543. 30920008

[bdr22456-bib-0016] Liu, S. , J. Rouleau , J. A. León , R. Sauve , K. S. Joseph , and J. G. Ray . 2015. “System, C. P. S. Impact of Pre‐Pregnancy Diabetes Mellitus on Congenital Anomalies, Canada.” Health Promotion and Chronic Disease Prevention in Canada: Research, Policy and Practice 35, no. 5: 79–84. 26186019 10.24095/hpcdp.35.5.01 PMC4910455

[bdr22456-bib-0017] Liu, S. , and S. W. Wen . 1999. “Development of Record Linkage of Hospital Discharge Data for the Study of Neonatal Readmission.” Chronic Diseases in Canada 20, no. 2: 77–81. 10455039

[bdr22456-bib-0018] Merhar, S. L. , J. E. Kline , A. Braimah , et al. 2021. “Prenatal Opioid Exposure Is Associated With Smaller Brain Volumes in Multiple Regions.” Pediatric Research 90, no. 2: 397–402. 10.1038/s41390-020-01265-w. 33177677 PMC8110593

[bdr22456-bib-0019] Miller, C. , D. Grynspan , L. Gaudet , et al. 2019. “Maternal and Neonatal Characteristics of a Canadian Urban Cohort Receiving Treatment for Opioid Use Disorder During Pregnancy.” Journal of Developmental Origins of Health and Disease 10, no. 1: 132–137. 10.1017/S2040174418000478. 30113278

[bdr22456-bib-0020] Nørgaard, M. , M. S. Nielsson , and U. Heide‐Jørgensen . 2015. “Birth and Neonatal Outcomes Following Opioid Use in Pregnancy: A Danish Population‐Based Study.” Substance Abuse: Research and Treatment 9, no. Suppl 2: 5–11. 10.4137/SART.S23547. PMC4599593 26512202

[bdr22456-bib-0021] Ordean, A. , S. Wong , and L. Graves . 2017. “No. 349‐Substance use in pregnancy.” Journal of Obstetrics and Gynaecology Canada 39, no. 10: 922–937. 10.1016/j.jogc.2017.04.028. 28935057

[bdr22456-bib-0022] Patrick, S. W. , J. Dudley , P. R. Martin , et al. 2015. “Prescription Opioid Epidemic and Infant Outcomes.” Pediatrics 135, no. 5: 842–850. 10.1542/peds.2014-3299. 25869370 PMC4411781

[bdr22456-bib-0023] Piske, M. , F. Homayra , J. E. Min , et al. 2021. “Opioid Use Disorder and Perinatal Outcomes.” Pediatrics 148, no. 4: e2021050279. 10.1542/peds.2021-050279. 34479983

[bdr22456-bib-0024] Plouffe, R. , V. Grywacheski , W. Luo , C. Nelson , and H. Orpana . 2023. “Neonatal Abstinence Syndrome Hospitalizations in Canada: A Descriptive Study.” Canadian Journal of Public Health 114, no. 2: 277–286. 10.17269/s41997-022-00726-5. 36482143 PMC9734797

[bdr22456-bib-0025] Public Health Agency of Canada . 2013. “Congenital Anomalies in Canada 2013: A Perinatal Health Surveillance Report.” Public Health Agency of Canada.

[bdr22456-bib-0026] Ramage, K. , K. Grabowska , C. Silversides , H. Quan , and A. Metcalfe . 2019. “Association of Adult Congenital Heart Disease With Pregnancy, Maternal, and Neonatal Outcomes.” JAMA Network Open 2, no. 5: e193667. 10.1001/jamanetworkopen.2019.3667. 31074818 PMC6512464

[bdr22456-bib-0027] Saleh Gargari, S. , M. Fallahian , L. Haghighi , M. Hosseinnezhad‐Yazdi , E. Dashti , and K. Dolan . 2012. “Maternal and Neonatal Complications of Substance Abuse in Iranian Pregnant Women.” Acta Medica Iranica 50, no. 6: 411–416. 22837120

[bdr22456-bib-0028] Short, T. D. 2019. “Gastroschisis Trends and Ecologic Link to Opioid Prescription Rates—United States, 2006–2015.” MMWR. Morbidity and Mortality Weekly Report 68, no. 2: 31–36. 10.15585/mmwr.mm6802a2. 30653484 PMC6336188

[bdr22456-bib-0029] Suarez, E. A. , B. T. Bateman , L. Straub , et al. 2024. “First Trimester Use of Buprenorphine or Methadone and the Risk of Congenital Malformations.” JAMA Internal Medicine 184, no. 3: 242–251. 10.1001/jamainternmed.2023.6986. 38252426 PMC10804281

[bdr22456-bib-0030] Suarez, E. A. , K. F. Huybrechts , L. Straub , et al. 2022. “Buprenorphine Versus Methadone for Opioid Use Disorder in Pregnancy.” New England Journal of Medicine 387, no. 22: 2033–2044. 10.1056/NEJMoa2203318. 36449419 PMC9873239

[bdr22456-bib-0031] Turner, S. D. , T. Gomes , X. Camacho , et al. 2015. “Neonatal Opioid Withdrawal and Antenatal Opioid Prescribing.” CMAJ Open 3, no. 1: E55–E61. 10.9778/cmajo.20140065. PMC4382031 25844370

[bdr22456-bib-0032] Viteri, O. A. , E. E. Soto , R. O. Bahado‐Singh , C. W. Christensen , S. P. Chauhan , and B. M. Sibai . 2015. “Fetal Anomalies and Long‐Term Effects Associated With Substance Abuse in Pregnancy: A Literature Review.” American Journal of Perinatology 32, no. 5: 405–416. 10.1055/s-0034-1393932. 25486291

[bdr22456-bib-0033] Wang, X. , Y. Wang , B. Tang , and X. Feng . 2022. “Opioid Exposure During Pregnancy and the Risk of Congenital Malformation: A Meta‐Analysis of Cohort Studies.” BMC Pregnancy and Childbirth 22: 401. 10.1186/s12884-022-04733-9. 35546223 PMC9097072

[bdr22456-bib-0034] Van der Weele, T. J. , and P. Ding . 2017. “Sensitivity Analysis in Observational Research: Introducing the E‐Value.” Annals of Internal Medicine 167, no. 4: 268–274. 10.7326/M16-2607. 28693043

[bdr22456-bib-0035] Wen, X. , N. Belviso , E. Murray , A. K. Lewkowitz , K. E. Ward , and K. J. Meador . 2021. “Association of gestational opioid exposure and risk of major and minor congenital malformations.” JAMA network open 4, no. 4: e215708. 33847750 10.1001/jamanetworkopen.2021.5708 PMC8044730

