# Supplementary material for: Prenatal Opioid Use Disorder and the Risk of Congenital Anomalies in Offspring: A Population‐Based Study
Source: Birth Defects Res. 2025 Feb 20;117(2):e2456. doi: 10.1002/bdr2.2456 (PMC11841020; doi:10.1002/bdr2.2456)
Supplement: Supplementary file 1 — Table S1. International Classification of Disease Codes for maternal risk factors, conditions or illnesses. Table S2. International Classification of Disease Codes: Infant Congenital Anomalies. Table S3. E‐values expressing the required risk ratio for any unmeasured confounder to overcome the observed association of specific congenital anomalies with prenatal diagnosis of opioid use disorder. [file BDR2-117-e2456-s001.docx]

Supplemental Table 1

International Classification of Disease Codes for maternal risk factors, conditions or illnesses

| Definition | ICD-10 CA codes |
| --- | --- |
| **Maternal substance use disorder diagnosis** |  |
| Opiate or opioids | F11, R78.1, P96.1, T40.0, T40.1, T40.02, T40.04, T40.06 |
| Tobacco | F17, T51.2, Z50.8, Z71.6, Z72.0 |
| Alcohol | F10, K70,Y15, X45, X65, K29.2, K86.0, K86.9, T51.0, T51.8, T51.9, R78.0,O35.4, Z50.2, Z50.8, Z71.4, Z72.1, Z86.40 |
| Cannabinoids | F12, T40.7 |
| Psychostimulants | F13, F14, F15, F16, R78.2, R82.5, T40.5 |
| Miscellaneous specified drugs | F18, F19, O35.5, R78.3, R78.4, R78.5, |
| Unspecified drugs | X42, X62, Y12-Y14, Z50.3, Z71.5, Z72.2, Z86.4 |
| **Maternal mental disorder** |  |
| Depression | F32, F34, F38, F39 |
| Stress and anxiety disorder | F40 – F48 |
| Bipolar disorder | F30, F31 |
| Schizophrenia and delusional disorders | F20 - F25, F28, F29 |
| Personality disorder | F60-F69 |
| **Maternal chronic illness and condition** |  |
| Epilepsy | G40, R75, T52, T60, X46, X48, X66, X68, Y16, Y18 |
| Lupus | L93.0-L93.2, M013, M32 |
| Obesity | E66 |
| Pregestational diabetes type 1, type 2 | E10, E11 |
| Migraine | G43 |
| Arthritis | M16.9, M17.9, M19.9 |
| Malignant neoplasms | C00 - C97 |
| Major non-chromosomal congenital anomalies | Q00.0 – Q89.9 |

Supplemental Table 2

International Classification of Disease Codes: Infant Congenital Anomalies

| Congenital anomalies |  | ICD-10 CA Codes |
| --- | --- | --- |
| Neural tube defects | Q01,Q01, Q03, Q05, Q07.0 | |
| Microcephaly | Q02 | |
| Critical congenital heart defects |  | |
| Hypoplastic left heart syndrome (HLHS) | Q23.4 | |
| Coarctation of the aorta (CoA) | Q25.1 | |
| Aortic valve stenosis (AoS) | Q23.0 | |
| Tetralogy of Fallot (TOF) | Q21.3 | |
| d-transposition of great arteries (DTGA) | Q20.3 | |
| Double outlet right ventricle (DORV) | Q20.1 | |
| Persistent truncus arteriosus (PTA) | Q20.0 | |
| Left ventricular outflow tract obstruction (LVOTO) | Q23.0, Q23.4, Q25.1 | |
| Conotruncal defects | Q20.0, Q20.1, Q20.3, Q21.3, Q252 | |
| Right ventricular outflow tract obstruction (RVOTO) | Q220, Q224 | |
| Ventricular septal defect (VSD) | Q21.0 | |
| Atrial septal defect (ASD) | Q21.1 | |
| Cleft palate | Q35 | |
| Cleft lip ± cleft palate | Q36, Q37 | |
| Esophageal atresia ± tracheoesophageal fistula | Q39.0, Q39.1 | |
| Anorectal atresia & stenosis | Q42 | |
| Hypospadias | Q54 | |
| Renal agenesis & stenosis | Q60 | |
| Cystic kidney disease | Q61 | |
| Transverse limb deficiency | Q71, Q72, Q73, Q74 | |
| Gastroschisis | Q79.3 | |

Supplemental Table 3.

E-values expressing the required risk ratio for any unmeasured confounder

to overcome the observed association of specific congenital anomalies

with prenatal diagnosis of opioid use disorder

| Exposure: diagnosed opioid use disorder | E-value | |
| --- | --- | --- |
|  | Point estimate | Lower confidence bound |
| Congenital microcephaly | 9.87 | 7.62 |
| Cleft palate | 8.89 | 6.88 |
| Atrial septal defect | 5.73 | 5.13 |
| Persistent truncus arteriosus | 4.78 | 1.31 |
| Ventricular septal defect | 4.46 | 3.74 |
| Coarctation of the aorta | 3.87 | 2.37 |
| Left ventricular outflow tract obstruction | 3.43 | 2.26 |
| Cystic kidney disease | 3.21 | 1.99 |
| Tetralogy of Fallot | 3.35 | 1.76 |
| Conotruncal defect | 3.15 | 2.04 |
| Neural tube defect | 2.64 | 1.46 |
|  |  |  |

The E-value of the point estimate is the minimum strength of association on the risk ratio scale

that an unmeasured confounder would need to have with both the exposure and the outcome,

conditional on the measured covariates, to fully explain away a specific exposure-outcome

association. The E-value of the lower confidence bound expresses the extent of unmeasured

confounding that would be required to shift the confidence interval so that it includes

a risk ratio of 1.00 (i.e., no association).
